# Impact of Comorbidities on Clinical Outcomes and Quality of Life of Patients With Hormone Receptor‐Positive/Human Epidermal Growth Factor Receptor 2‐Negative (HR+/HER2−) Advanced Breast Cancer Treated With Palbociclib in the POLARIS Study

**DOI:** 10.1002/cam4.71788

**Published:** 2026-04-22

**Authors:** Debu Tripathy, Joanne L. Blum, Meghan S. Karuturi, Steven McCune, Sobha Kurian, Mehdi M. Moezi, Daniel M. Anderson, Yan Ji, Timothy J. Pluard, John Migas, Shailendra Lakhanpal, Erin Jepsen, Yao Wang, Monica Z. Montelongo, Zhe Zhang, Joseph C. Cappelleri, Eric Gauthier, Gabrielle B. Rocque

**Affiliations:** ^1^ Department of Breast Medical Oncology The University of Texas MD Anderson Cancer Center Houston Texas USA; ^2^ Department of Oncology, Baylor‐Sammons Cancer Center Texas Oncology, US Oncology Dallas Texas USA; ^3^ William S. Gibbons Cancer Research Institute Northwest Georgia Oncology Centers Wellstar Marietta Georgia USA; ^4^ Hematology/Oncology West Virginia University Cancer Institute Morgantown West Virginia USA; ^5^ Medical Oncology Cancer Specialists of North Florida Fleming Island Florida USA; ^6^ Oncology & Hematology Health Partners Institute Saint Paul Minnesota USA; ^7^ Health Partners Institute St. Paul Minnesota USA; ^8^ Saint Luke's Cancer Institute Kansas City Missouri USA; ^9^ Mid‐Illinois Hematology & Oncology Associates Ltd Normal Illinois USA; ^10^ Saint Vincent's Birmingham Birmingham Alabama USA; ^11^ Novant Health Winston‐Salem North Carolina USA; ^12^ Oncology Medical Group Pfizer Inc New York New York USA; ^13^ Biostatistics and Medical Writing, ICON Commercialisation and Outcomes, ICON Plc. Blue Bell Pennsylvania USA; ^14^ Oncology Clinical Statistics Pfizer Inc San Diego California USA; ^15^ Statistical Research and Data Science Center Pfizer Inc Groton Connecticut USA; ^16^ Global Medical Affairs Pfizer Inc San Francisco California USA; ^17^ Division of Hematology/Oncology, Department of Medicine University of Alabama at Birmingham Birmingham Alabama USA

**Keywords:** clinical outcomes, comorbidities, palbociclib, quality‐of‐life, real‐world, treatment patterns

## Abstract

**Background:**

Comorbidities, common in patients with advanced breast cancer (ABC), may impact survival outcomes and health‐related quality of life (HRQoL). Here, we report subgroup analyses on the basis of comorbidities of patients from POLARIS (NCT03280303), a prospective, observational study of patients with hormone receptor‐positive/human epidermal growth factor receptor 2‐negative (HR+/HER2−) ABC who were treated with palbociclib plus endocrine therapy in routine clinical practice in North America.

**Methods:**

Real‐world progression‐free survival (rwPFS) and overall survival (OS) were evaluated by line of therapy (1 LOT, ≥ 2 LOT), Charlson Comorbidity Index (CCI) score (0, 1–2, and 3+), and common comorbid disorder categories (cardiovascular, psychiatric, metabolic and nutritional, and blood and lymphatic disorders). HRQoL was assessed using the global health status (GHS)/QoL subscale of the European Organization for Research and Treatment of Cancer Quality‐of‐Life Questionnaire Core 30.

**Results:**

From January 2017 to October 2019, 1250 patients (median age, 64 years) initiated palbociclib‐based therapy. Median rwPFS (95% CI) for patients with CCI scores of 0, 1–2, and 3+ were 20.3 (17.1–24.8), 24.2 (19.4–29.5), and 16.8 (11.2–20.8) months in 1 LOT and 13.7 (6.2–19.7), 13.2 (9.4–17.5), and 14.9 (8.0–21.9) months in ≥ 2 LOT, respectively. Median OS durations (95% CI) were 48.8 (37.3–not estimable [NE]), not reached (43.0–NE), and 34.8 (29.1–44.1) months in 1 LOT and 39.0 (30.6–50.5), 37.9 (26.5–42.6), and 31.6 (20.9–45.2) months in ≥ 2 LOT, respectively. Patients with blood and lymphatic disorders had shorter rwPFS and OS than those with other common comorbidities. GHS/QoL was maintained irrespective of CCI score. Patients with a CCI score of 0 had clinically meaningful and statistically significantly higher mean GHS/QoL scores than patients with a CCI score of 3+ at each assessment.

**Conclusions:**

Patients with HR+/HER2− ABC receiving palbociclib with higher comorbidity burden, especially blood and lymphatic system disorders, had poorer clinical outcomes. GHS/QoL was preserved regardless of comorbidity burden.

**Trial Registration:**

Clinical trial number: NCT03280303

**Précis**

This real‐world study evaluated the impact of comorbidities on clinical outcomes and HRQoL for patients with HR+/HER2– ABC who were treated with palbociclib plus endocrine therapy. Poorer rwPFS and OS were observed for patients who had a high comorbidity burden (CCI 3+), a population that is frequently underrepresented in RCTs; on‐treatment global HRQoL was maintained irrespective of CCI score.

## 1 Introduction

Comorbidities are common in patients with breast cancer [1], and can have a considerable impact on the patient journey. The presence of comorbid disorders is generally associated with poor survival outcomes [1, 2, 3, 4], and can influence cancer treatment decision‐making, concomitant medication usage, and other factors [5, 6]. Patient quality of life (QoL), a crucial consideration for non‐curative treatment strategies, is also negatively impacted by comorbidities [6, 7]. Despite the myriad interactions between comorbid disorders and breast cancer, relatively few studies have explored the impact of comorbidities on outcomes in the advanced/metastatic breast cancer (ABC/MBC) setting.

Since the approval of palbociclib as the first‐in‐class cyclin‐dependent kinase (CDK) 4/6 inhibitor in 2015 [8], CDK4/6 inhibitors, in combination with endocrine therapy (ET), have become the standard of care for patients with hormone receptor‐positive/human epidermal growth factor receptor 2‐negative (HR+/HER2−) ABC/MBC [9, 10]. A series of phase 3 randomized controlled trials (RCTs) demonstrated that the addition of a CDK4/6 inhibitor to ET prolonged progression‐free survival (PFS) in first‐ and later‐line settings [11, 12, 13, 14, 15, 16, 17]. Although the gold standard for assessing treatment efficacy, RCTs generally feature restrictive eligibility criteria that often exclude patients who are being treated for various chronic conditions [18]. As a result, healthcare providers, regulatory bodies, and payers may lack crucial efficacy data for some patient subpopulations. Real‐world studies complement clinical trials by evaluating outcomes among the more heterogeneous patient population treated in routine clinical practice [19, 20]. For example, a large‐scale (*N = 2888*), real‐world study using the Flatiron database showed that palbociclib plus ET was associated with longer real‐world PFS (rwPFS) and overall survival (OS) than ET alone in patients with HR+/HER2− MBC, though outcomes were not assessed by comorbid disease burden [21].

Despite the growing body of literature describing the effectiveness of palbociclib plus ET in the real‐world setting, the impact of comorbidities on real‐world clinical outcomes for patients receiving palbociclib and other CDK4/6 inhibitors remains largely unknown. Furthermore, data describing how palbociclib treatment and the presence of comorbidities impact patient health‐related QoL (HRQoL) are scarce. The POLARIS study employed a prospective, longitudinal, non‐interventional design to assess palbociclib real‐world clinical outcomes, HRQoL, and treatment patterns in patients with HR+/HER2− ABC [22, 23], including those in subgroups often excluded or underrepresented in RCTs, such as patients with comorbidities. Here, we describe rwPFS, OS, and HRQoL in patients with HR+/HER2− ABC/MBC and comorbid disorders who were treated with palbociclib in routine clinical practice in the POLARIS study.

## 2 Methods

### 2.1 Study Design

POLARIS (NCT03280303) is a prospective, non‐interventional, multicenter, real‐world cohort study of patients aged ≥ 18 years with HR+/HER2− ABC/MBC treated with palbociclib in routine clinical practice from > 100 sites in the United States and Canada. Details on study design and methods have been reported previously [22]. Briefly, inclusion criteria were a diagnosis of ABC/MBC, documented HR+ and HER2− tumor status, and a candidate to receive palbociclib treatment as determined by the treating physician. Exclusion criteria were a life expectancy of < 3 months at the time of ABC/MBC diagnosis, participation in an interventional clinical trial, and active treatment for malignancies other than ABC/MBC at the time of enrollment. Demographic, treatment, disease assessment, comorbidities, and longitudinal HRQoL data were collected via electronic case report forms from the treating physicians' routine clinical assessments per their local standard of care. Comorbidities or comorbid conditions were defined as medical conditions concurrent with MBC, including those in the Charlson Comorbidity Index (CCI) plus leukemia, lymphoma, neutropenia, and anemia (Table S1). Clinical data, including current comorbid disorders, were coded using the Medical Dictionary for Regulatory Activities (MedDRA) v19.1. Patients were followed from the start of palbociclib treatment (between January 2017 and October 2019) until approximately 3 years after the end of palbociclib treatment, patient withdrawal from the study, or death, whichever came first. Written informed consent was obtained from all patients before study enrollment. The study was conducted in accordance with all applicable legal and regulatory requirements, the protection of patient personal data was ensured by all parties, and the study was prospectively approved by the appropriate institutional review boards or independent ethics committees.

### 2.2 Outcomes

rwPFS was defined as time from initiation of study treatment until physician‐documented disease progression (based on imaging, tissue biopsy, biomarkers, or clinical judgment), or death due to any cause, whichever occurred first, during a patient's respective line of therapy (LOT, 1 L or ≥ 2 LOT). Patients who did not have documented disease progression or death were censored at the last date of response assessment without progressive disease through the start date of the next LOT (for patients with ≥ 1 LOT after palbociclib). Patients who did not receive a subsequent LOT after palbociclib were censored at their last date of response assessment without progressive disease during the study period. OS was defined as the time from initiation of study treatment to date of death due to any cause; patients who did not have a documented death were censored at the last available visit date.

HRQoL was assessed by the European Organization for Research and Treatment of Cancer Quality‐of‐Life Questionnaire Core 30 (EORTC QLQ‐C30) [24]. The EORTC QLQ‐C30 is a 30‐item questionnaire that yields functional, symptom, and global health status (GHS)/QoL subscale scores. GHS/QoL scores were derived from two 7‐point Likert scale items reporting patient overall health and QoL from “very poor” to “excellent.” Raw scores from the GHS/QoL items were converted to a 0 to 100 scale, with higher scores representing a better level of GHS/QoL [24, 25, 26]. The EORTC QLQ‐C30 tool was administered at baseline and monthly for the first 3 months of treatment with palbociclib, then every 3 months until the end of treatment. Mean GHS/QoL was calculated at baseline, 6, 12, and 18 months. Missing data were imputed according to EORTC guidelines, with a domain considered missing if > 50% of items were missing.

### 2.3 Statistical Analysis

The analysis population consisted of all enrolled patients who received at least 1 dose of palbociclib as of the data cutoff (overall population). A secondary per‐label population was also analyzed; this subgroup consisted of patients in the overall population who received palbociclib plus an aromatase inhibitor as 1 L treatment or palbociclib plus fulvestrant after prior ET in any setting as per the approved US label indications [27]. Descriptive statistics were used to present palbociclib treatment patterns (starting dose, dose modification, dose decrease, and dosing interruption) and patient characteristics. For rwPFS and OS, medians and associated confidence intervals (CIs) were estimated using the Kaplan–Meier survival method [28]. Median follow‐up duration was calculated using the reverse Kaplan–Meier estimator [29]. For the time‐to‐event analyses, no hypothesis testing was performed; all analyses were descriptive.

rwPFS, OS, and HRQoL patient‐reported outcomes (PROs) were analyzed by prespecified subgroups, the CCI classification [30], and LOT. The CCI score was calculated by assigning a point score for each baseline comorbidity disorder (Table S1), then summing up all points for each patient to generate an overall score, with a higher score indicating a more severe comorbidity burden [30]. For our analysis, CCI scores were classified as 0, 1–2, and 3+. LOT was defined as the number of systemic therapies received after initial ABC/MBC diagnosis but preceding initiation of palbociclib treatment. Patients receiving 1 LOT had no prior LOT in the ABC/MBC setting before starting palbociclib, and those receiving ≥ 2 LOT had ≥ 1 prior LOT in the ABC/MBC setting before starting palbociclib. Post hoc analyses also evaluated rwPFS and OS by the 4 most common CCI comorbid disorders (documented in > 15% of patients) present in the dataset: Cardiovascular disorders (congestive heart failure, hypertension, history of myocardial infarction, and peripheral vascular disease), psychiatric disorders (depression), blood and lymphatic system disorders (anemia and neutropenia), and metabolic and nutritional disorders (diabetes). Although not included in the CCI, anemia and neutropenia histories were collected by site investigators.

Completion rates of the EORTC QLQ‐C30 GHS/QoL subscale (percentage calculated by dividing the number of patients who completed at least 50% of the items by the total number of patients who received at least 1 dose of palbociclib) and GHS/QoL mean scores were determined at baseline and months 6, 12, and 18. A higher score indicated better perceived GHS/QoL. Baseline GHS/QoL scores were compared to US population normative data [31]. Preplanned pairwise comparisons of mean GHS/QoL scores between CCI subgroups were conducted at each time point, with a clinically meaningful difference defined as ≥ 10 points [32]. Statistical significance of such differences was analyzed using independent sample *t*‐tests [25, 28]. Mean change from baseline in GHS/QoL mean score at months 6, 12, and 18 was also determined and evaluated. Statistical significance of change from baseline was evaluated using paired *t*‐tests [25, 28].

## 3 Results

### 3.1 Patients

The POLARIS study enrolled 1285 patients in total, and the study population included 1250 patients who received at least 1 dose of palbociclib between January 4, 2017, and October 3, 2019. The data cutoff date was January 9, 2023. Median patient age was 64 years (range, 22–97 years), 98.8% were female, 27.3% had de novo MBC at study enrollment. In patients with MBC at enrollment (*n = 1186*), 34.1% had bone‐only disease, and 41.7% had visceral disease (Table 1). Median baseline total comorbidity burden (Charlson Index comorbidities plus hematologic and other comorbidities listed by site investigators at the time of enrollment and held constant over the course of the study) was 2 (range, 0–9) comorbidities; 5.0% of patients had 0 comorbidities, 56.0% had 1–2, 32.3% had 3–4, and 6.6% had > 4.

**TABLE 1:** Baseline demographic and disease characteristics by CCI.

	CCI 0 (*n* = 377)	CCI 1–2 (*n* = 682)	CCI 3+ (*n* = 191)	Total (*N* = 1250)
Age at study enrollment, years				
No. (missing)	376 (1)	679 (3)	191 (0)	1246 (4)
Mean (SD)	57.4 (12.6)	64.8 (12.1)	67.6 (10.4)	63.0 (12.6)
Median (range)	58.0 (22–97)	66.0 (29–97)	68.0 (40–87)	64.0 (22–97)
Female sex, No. (%)	375 (99.5)	672 (98.5)	188 (98.4)	1235 (98.8)
Race, No. (%)				
White	297 (78.8)	569 (83.4)	156 (81.7)	1022 (81.8)
Black or African American	38 (10.1)	75 (11.0)	26 (13.6)	139 (11.1)
Asian	13 (3.4)	7 (1.0)	3 (1.6)	23 (1.8)
American Indian or Alaska Native	2 (0.5)	4 (0.6)	2 (1.0)	8 (0.6)
Native Hawaiian or other Pacific Islander	2 (0.5)	2 (0.3)	1 (0.5)	5 (0.4)
Other	10 (2.7)	11 (1.6)	1 (0.5)	22 (1.8)
Not reported or missing	15 (4.0)	14 (2.1)	2 (1.0)	31 (2.5)
Ethnicity, No. (%)				
Not Hispanic or Latino	326 (86.5)	609 (89.3)	171 (89.5)	1106 (88.5)
Hispanic or Latino	33 (8.8)	54 (7.9)	19 (9.9)	106 (8.5)
Not reported or missing	18 (4.8)	19 (2.8)	1 (0.5)	38 (3.0)
Time since ABC/MBC diagnosis, months				
No. (missing)	377 (0)	678 (4)	191 (0)	1246 (4)
Mean (SD)	11.9 (27.0)	12.5 (26.7)	17.4 (37.2)	13.1 (28.7)
Median (range)	1.4 (0–191)	1.2 (0–242)	1.4 (0–248)	1.3 (0–248)
Disease stage at enrollment, No. (%)				
Locally advanced (stage III)	21 (5.6)	28 (4.1)	13 (6.8)	62 (5.0)
Metastatic (stage IV)	356 (94.4)	652 (95.6)	178 (93.2)	1186 (94.9)
Not reported	0	2 (0.3)	0	2 (0.2)
Disposition at enrollment, No. (%)				
Recurrent from earlier stage	258 (68.4)	463 (67.9)	128 (67.0)	849 (67.9)
De novo, newly diagnosed stage IV	95 (25.2)	190 (27.9)	56 (29.3)	341 (27.3)
Not reported	24 (6.4)	29 (4.3)	7 (3.7)	60 (4.8)
Bone metastases at MBC diagnosis^a^, No. (%)				
Bone plus other	149 (41.9)	252 (38.7)	80 (44.9)	481 (40.6)
Bone only	112 (31.5)	242 (37.1)	51 (28.7)	405 (34.1)
Visceral metastases^b^ at MBC diagnosis^a^, No. (%)				
Yes	158 (44.4)	256 (39.3)	80 (44.9)	494 (41.7)
No	198 (55.6)	396 (60.7)	98 (55.1)	692 (58.3)

Abbreviations: ABC, advanced breast cancer; CCI, Charlson Comorbidity Index; MBC, metastatic breast cancer; SD, standard deviation.

^a^ Among patients with MBC at study enrollment.

^b^ Visceral disease refers to metastases of the brain, liver, and/or lung/pleura.

Baseline demographic and disease characteristics by CCI score are presented in Table 1; 30.2% of patients had a CCI score of 0, 54.6% had a score of 1–2, and 15.3% had a score of 3+. Baseline demographic and disease characteristics are also presented by comorbid disorder in Table S2. Overall, 690 patients (55.2%) had cardiovascular disorders, whereas 332 (26.6%), 230 (18.4%), and 228 (18.2%) had psychiatric disorders, blood and lymphatic system disorders, and metabolic/nutritional disorders, respectively. Palbociclib was given as 1 LOT and ≥ 2 LOT in 901 and 349 patients, respectively (Table 2).

**TABLE 2:** Palbociclib treatment patterns by CCI and LOT.

1 LOT (*n* = 901) CCI		0	1–2	3+
	No.	*n* = 274	*n* = 489	*n* = 138
Starting dose, No. (%)				
125 mg	827	258 (94.2)	451 (92.2)	118 (85.5)
100 mg	53	9 (3.3)	28 (5.7)	16 (11.6)
75 mg	21	7 (2.6)	10 (2.0)	4 (2.9)
Dose modification, No. (%)				
No	551	177 (64.6)	295 (60.3)	79 (57.2)
≥ 1	350	97 (35.4)	194 (39.7)	59 (42.8)
Dose decrease, No. (%)				
No	559	178 (65.0)	299 (61.1)	82 (59.4)
≥ 1	342	96 (35.0)	190 (38.9)	56 (40.6)
Dosing interruption, No. (%)				
No	761	226 (82.5)	414 (84.7)	121 (87.7)
≥ 1	140	48 (17.5)	75 (15.3)	17 (12.3)

≥ 2 LOT (*n* = 349)				
CCI		0	1–2	3+
		*n* = 103	*n* = 193	*n* = 53
Starting dose, No. (%)				
125 mg	297	86 (83.5)	169 (87.6)	42 (79.2)
100 mg	39	15 (14.6)	15 (7.8)	9 (17.0)
75 mg	13	2 (1.9)	9 (4.7)	2 (3.8)
Dose modification, No. (%)				
No	223	65 (63.1)	124 (64.2)	34 (64.2)
≥ 1	126	38 (36.9)	69 (35.8)	19 (35.8)
Dose decrease, No. (%)				
No	227	68 (66.0)	125 (64.8)	34 (64.2)
≥ 1	122	35 (34.0)	68 (35.2)	19 (35.8)
Dosing interruption, No. (%)				
No	300	88 (85.4)	170 (88.1)	42 (79.2)
≥ 1	49	15 (14.6)	23 (11.9)	11 (20.8)

Abbreviations: CCI, Charlson Comorbidity Index; LOT, line of therapy.

### 3.2 Clinical Outcomes by CCI Score

**FIGURE 1:**
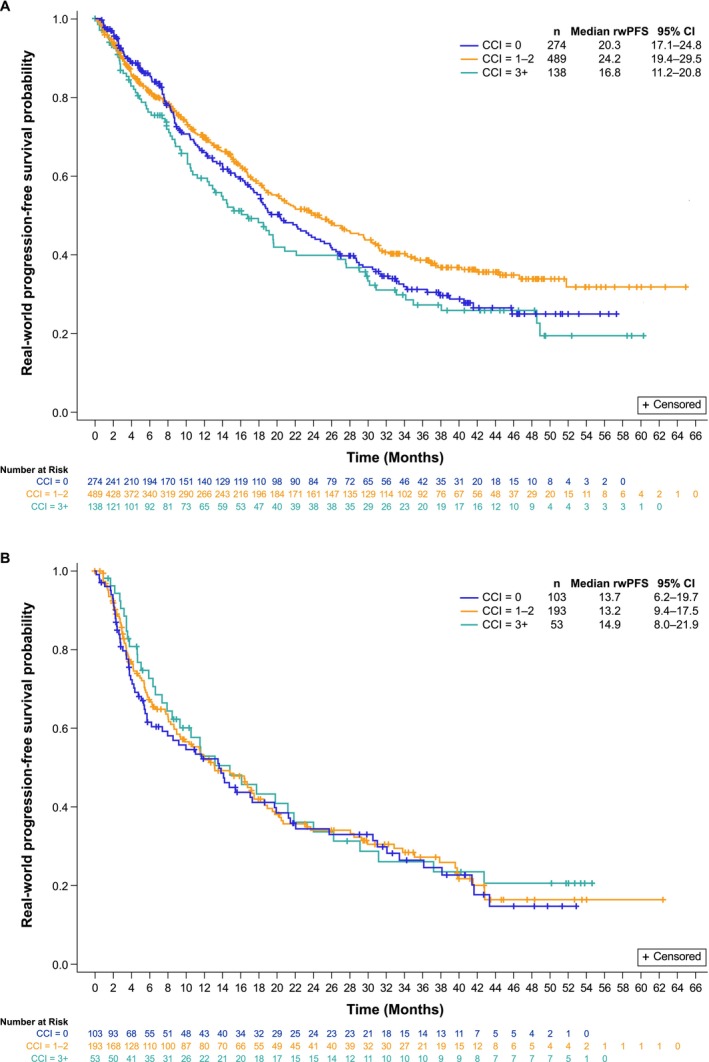
rwPFS by CCI score in the 1 LOT (A) and the ≥ 2 LOT (B). CCI, Charlson Comorbidity Index; CI, confidence interval; LOT, line of therapy; rwPFS, real‐world progression‐free survival.

**FIGURE 2:**
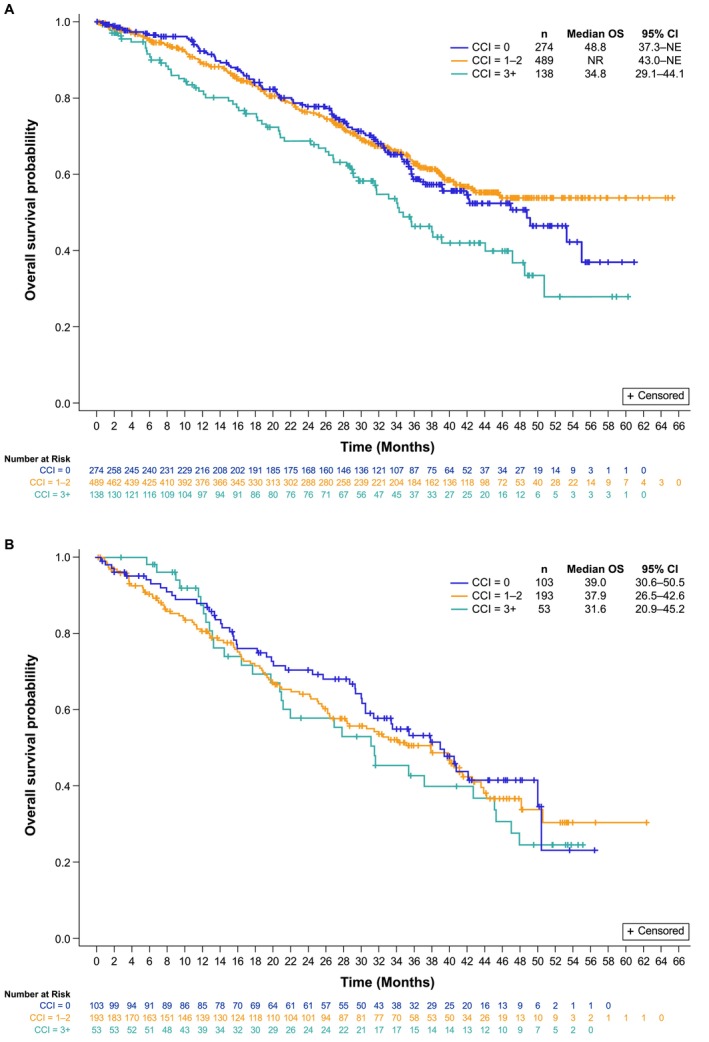
OS by CCI in the 1 LOT (A) and the ≥ 2 LOT (B). CCI, Charlson Comorbidity Index; CI, confidence interval; LOT, line of therapy; NE, not estimable; NR, not reached; OS, overall survival.

rwPFS and OS are summarized in Figures 1 and 2, respectively, by LOT and CCI score. After a median follow‐up of 35.4 (95% CI, 33.1–37.5) months, in patients receiving palbociclib as 1 L, median rwPFS was numerically shorter for patients with CCI 3+ score (16.8 [95% CI, 11.2–20.8] months) than those with CCI ≤ 2 score (24.2 [95% CI, 19.4–29.5] and 20.3 [95% CI, 17.1–24.8] months for CCI 1–2 and CCI 0, respectively; Figure 1A). In patients treated in the ≥ 2 L setting, after a median follow‐up of 35.8 (95% CI, 31.6–40.6) months, similar median rwPFS was observed irrespective of CCI score (13.7 [95% CI, 6.2–19.7], 13.2 [95% CI, 9.4–17.5], and 14.9 [8.0–21.9] months for patients with CCI 0, 1–2, and 3+, respectively, Figure 1B).

Regardless of LOT, median OS tended to be numerically shorter in patients with a CCI 3+ score than those with a CCI ≤ 2 score. With a median follow‐up of 38.6 (95% CI, 37.3–40.0) months, median OS for patients with CCI 3+ receiving 1 L palbociclib was 34.8 (95% CI, 29.1–44.1) months compared with 48.8 (95% CI, 37.3–not estimable [NE]) months and not reached (95% CI, 43.0–NE) for patients with CCI 0 and 1–2, respectively (Figure 2A). Patients treated with ≥ 2 L palbociclib, after a median follow‐up of 41.0 (95% CI, 38.1–42.6) months, median OS for patients with CCI 3+ was 31.6 (95% CI, 20.9–45.2) months compared with 39.0 (95% CI, 30.6–50.5) and 37.9 (95% CI, 26.5–42.6) months for patients with CCI 0 and 1–2, respectively (Figure 2B).

### 3.3 Clinical Outcomes by Comorbid Disorder

**FIGURE 3:**
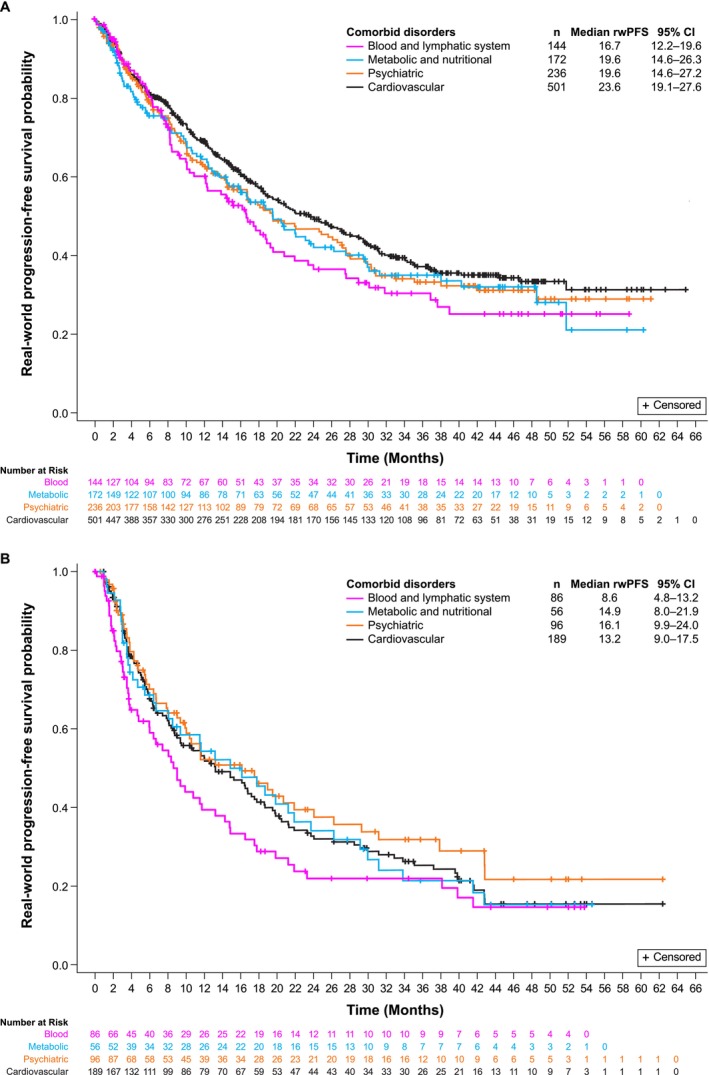
RwPFS by comorbid disorder in the 1 LOT (A) and the ≥ 2 LOT (B). CI, confidence interval; LOT, line of therapy; rwPFS, real‐world progression‐free survival.

**FIGURE 4:**
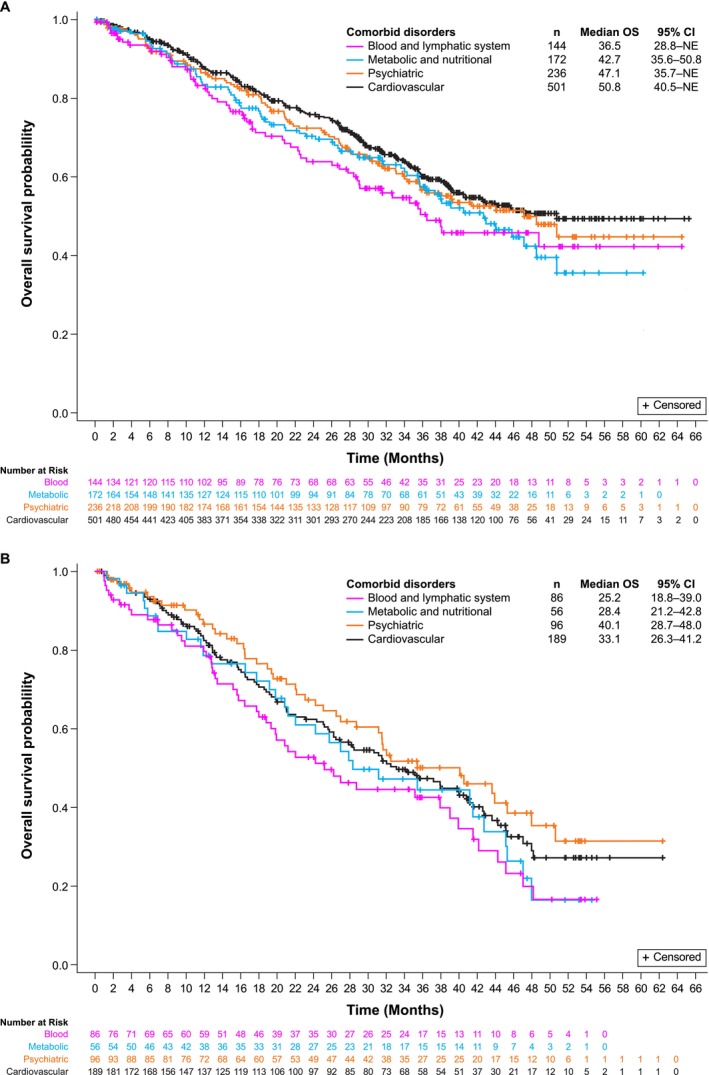
OS by comorbid disorder in the 1 LOT (A) and the ≥ 2 LOT (B). CI, confidence interval; LOT, line of therapy; OS, overall survival.

rwPFS and OS are summarized in Figures 3 and 4, respectively, by LOT and comorbid disorder. Median rwPFS was numerically shorter in patients with blood and lymphatic system disorders relative to those with other common comorbidities, regardless of LOT. For patients treated in the 1 L, median rwPFS was 16.7 (95% CI, 12.2–19.6) months for the blood and lymphatic system disorders group compared with 19.6 (95% CI, 14.6–26.3), 19.6 (95% CI, 14.6–27.2) and 23.6 (95% CI, 19.1–27.6) months for the metabolic and nutritional, psychiatric, and cardiovascular disorders groups, respectively (Figure 3A). In the ≥ 2 L setting, median rwPFS for the blood and lymphatic disorders group was 8.6 (95% CI, 4.8–13.2) months compared with 14.9 (95% CI, 8.0–21.9), 16.1 (95% CI, 9.9–24.0) and 13.2 (95% CI, 9.0–17.5) months for the metabolic and nutritional, psychiatric, and cardiovascular disorders groups, respectively (Figure 3B). Similarly, regardless of LOT, median OS was numerically shorter in patients with blood and lymphatic system disorders relative to those with other common comorbidities (Figure 4).

### 3.4 GHS/QoL Scores by CCI

**FIGURE 5:**
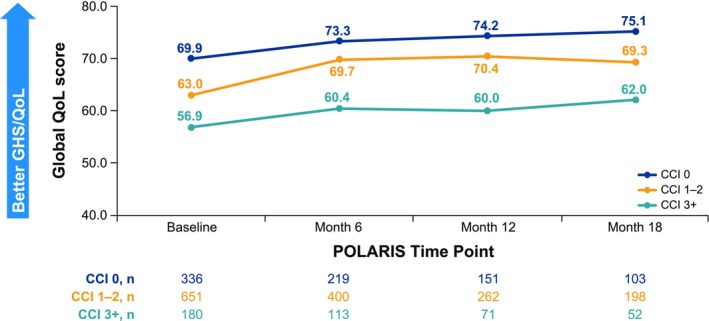
Mean GHS/QoL scores by CCI. CCI, Charlson Comorbidity Index; GHS, global health status; QoL, quality of life.

**FIGURE 6:**
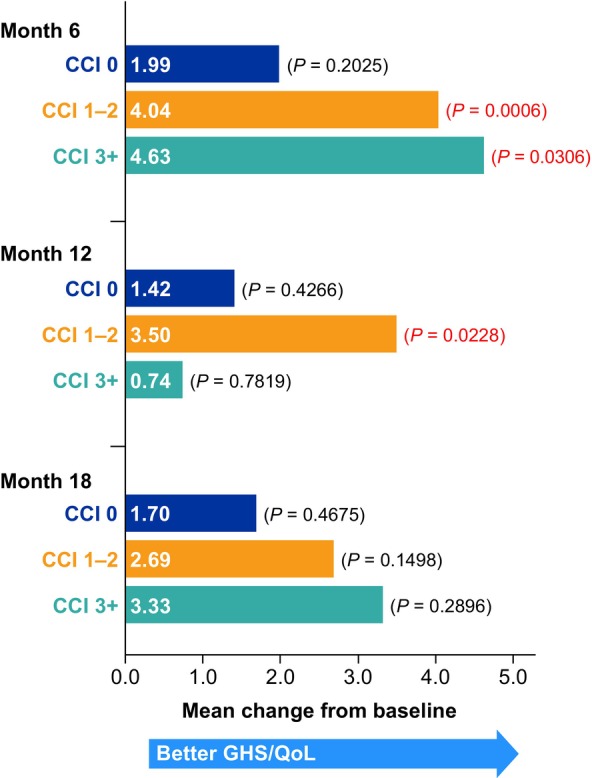
GHS/QoL scores, mean change from baseline over time by CCI. Mean change from baseline based on all available patients who had data at both baseline and at months 6, 12, and 18. Month 6, sample sizes: CCI 0, 219; CCI 1–2, 400; CCI ≥ 3, 113. Month 12, sample sizes: CCI 0, 151; CCI 1–2, 262; CCI ≥ 3, 71. Month 18, sample sizes: CCI 0, 103; CCI 1–2, 198; CCI ≥ 3, 52. CCI, Charlson Comorbidity Index; GHS, global health status; QoL, quality of life.

In the overall population, the completion rate of the GHS/QoL domain on the EORTC QLQ‐C30 was 93.4% at baseline, which decreased at 6‐month (58.6%), 12‐month (38.7%), and 18‐month (28.2%) assessments; completion rates were generally similar across CCI score groups at each time point (Table S3). Mean baseline GHS/QoL scores did not differ from general population normative data (65.6 for women aged 60–69 years) [31] by a magnitude deemed clinically meaningful (≥ 10 points) [32] for any of the CCI groups (CCI 0, 69.9; CCI 1–2, 63.0; CCI 3+, 56.9, Figure 5). In most instances, statistically significant differences in mean GHS/QoL scores were observed between CCI groups at baseline as well as at each time point assessed (Table 3). Patients with CCI 0 had clinically meaningful higher mean GHS/QoL scores than patients with CCI 3+ at baseline, 6‐, 12‐, and 18‐month assessments, and patients with CCI 1–2 had a clinically meaningful higher mean GHS/QoL score than those with CCI 3+ at the 12‐month assessment (Table 3, Figure 5). For patients with GHS/QoL data at both baseline and each follow‐up assessment time point, numerical improvements in GHS/QoL were observed. However, despite these improvements being statistically significant in some cases, they did not reach the clinically meaningful threshold (Figure 6).

**TABLE 3:** GHS/QoL score comparisons among CCI groups.

GHS/QoL	CCI 0 vs. 1–2		CCI 0 vs. 3+		CCI 1–2 vs. 3+	
	Diff	*p‐*value	Diff	*p*‐value	Diff	*p*‐value
Baseline	6.94	< 0.0001	**12.99**	< 0.0001	6.06	0.0028
Month 6	3.60	0.0401	**12.89**	< 0.0001	9.29	< 0.0001
Month 12	3.81	0.0546	**14.25**	< 0.0001	**10.44**	0.0002
Month 18	5.80	0.0225	**13.06**	0.0004	7.26	0.0314

*Note:* Mean absolute Global QoL based on all available patients at any time point. Higher scores are more favorable. Bold font indicates a ≥ 10‐point difference, indicative of a clinically meaningful difference. GHS/QoL scores at baseline and months 6, 12, and 18 are provided in Figure 5.

Abbreviations: CCI, Charlson Comorbidity Index; Diff, difference; GHS, global health status; QoL, quality of life.

### 3.5 Treatment Patterns

In the overall population, 1124 (89.9%) patients started at the label‐recommended starting dose of palbociclib 125 mg/day [27]; of the 126 (10.1%) patients who started palbociclib at a lower dose (100 or 75 mg), 18 (1.4%) did so due to existing comorbidities. Other reasons for starting at a reduced palbociclib dose were age (3.2%), previous treatment (1.0%), or “other” (4.0%). In the 1 L setting, most patients (91.8%) initiated palbociclib at 125 mg/day; patients with CCI 3+ were more likely to initiate palbociclib at a lower dose (14.5%) compared with patients with CCI 1–2 (7.8%) and CCI 0 (5.8%) (Table 2, Figure S1). In the ≥ 2 L setting, a lower proportion of patients initiated palbociclib at 125 mg/day (85.1%) than those receiving palbociclib in the 1 L setting (91.8%). Similar to the pattern observed in 1 L, in ≥ 2 L, a higher percentage of patients with CCI 3+ initiated palbociclib at reduced doses (20.8%) than those in the CCI 1–2 (12.4%) and CCI 0 (16.5%) groups. In both 1 L and ≥ 2 L settings, a lower percentage (81.7%) of patients in the blood and lymphatic system disorders group initiated palbociclib 125 mg/day compared with patients in the other comorbidity groups (87.3%, 89.9%, and 90.1% for metabolic/nutritional, cardiovascular, and psychiatric disorders, respectively) (Table S4). In both 1 L and ≥ 2 L settings, the frequency of dose modifications, dose decreases, and dosing interruptions was broadly similar across different comorbidity groups (Table S4).

### 3.6 Clinical Outcomes and PROs: Per‐Label Analysis Set

Clinical outcomes and PROs were also assessed in the per‐label analysis set (*n = 861*), wherein 712 patients were treated as 1 L, and 149 patients were treated as ≥ 2 L. Baseline demographic and disease characteristics are presented by CCI and by comorbidity in Tables S5 and S6, respectively. In the CCI groups, survival outcomes for patients treated in the 1 L setting were broadly consistent with the overall population (Figures S2 and S3). In the ≥ 2 L setting, per‐label treated patients in the CCI 1–2 group had worse outcomes than in the overall population, but sample sizes were small. Irrespective of LOT, trends in survival outcomes across comorbidity subgroups were generally similar between the overall population and the per‐label analysis set (Figures S4 and S5). As in the overall population, GHS/QoL was maintained at all time points for patients regardless of CCI score (Figures S6 and S7).

## 4 Discussion

In this prospective, real‐world study of patients with HR+/HER2− ABC/MBC treated with palbociclib plus ET, comorbidities were common, and higher comorbidity burden was associated with generally poorer clinical outcomes, lower HRQoL at baseline and on‐treatment, and differences in palbociclib treatment patterns. Patients with a CCI score of 3+ tended to have shorter rwPFS and OS than those with CCI scores of ≤ 2 in the 1 L setting. On the other hand, patients with moderate comorbidity burden (CCI 1–2) had clinical outcomes generally similar to those without documented comorbidities. Although the CCI 1–2 group had numerically better outcomes than the CCI 0 group, these advantages were neither significant nor clinically meaningful. The CCI 0 group was younger, with slightly higher tumor burden and more visceral involvement than the CCI 1–2 group, possibly indicating more aggressive disease in these patients. In the ≥ 2 L setting, CCI subgroups had similar median rwPFS, but patients with a CCI score 3+ had shorter OS than those with CCI scores ≤ 2; however, the relatively small size of the CCI 3+ group and the shorter expected PFS and OS of this heavily pretreated population preclude robust conclusions.

The CCI score (0, 1–2, and 3+) was selected as the primary, prespecified subgroup analysis for this study. While the CCI provides a summary score reflecting a patient's level of comorbidity, it does not convey information about specific systems directly affected by each comorbid condition, nor the medications that might typically be used to manage these conditions. Therefore, we categorized individual comorbidities into groups based on the primary systems affected and conducted post hoc survival analyses on the 4 comorbidity category subgroups that were most prevalent in the study population (cardiovascular, psychiatric, blood and lymphatic, and metabolic and nutritional disorders). Irrespective of LOT, patients with blood and lymphatic system disorders had numerically shorter rwPFS and OS than those with other comorbidities. Anemia and neutropenia were 2 specific comorbid disorders commonly reported within this subgroup. Anemia, common in patients with breast cancer, can negatively impact treatment efficacy, patient QoL, and survival [33]. Patients with preexisting lower baseline absolute neutrophil count are significantly more likely to experience grade 3/4 neutropenia while receiving palbociclib, potentially contributing to treatment disruption and poor clinical outcomes [34]. Notably, anemia and neutropenia are not included in the CCI, highlighting the importance of evaluating the impact of certain additional comorbidities on ABC/MBC outcomes. Patients in the other 3 comorbidity categories treated in the 1 L setting had median rwPFS and OS similar to the overall POLARIS population (20.9 [95% CI, 18.7–24.7] months and 48.5 [95% CI, 42.0–NE] months, respectively) [23]. In a post hoc analysis of PALOMA‐2, palbociclib plus letrozole significantly prolonged median PFS compared with placebo plus letrozole in each of the preexisting condition subgroups examined: Gastrointestinal, musculoskeletal, metabolic, vascular/cardiac, and vascular only [35].

In this study, baseline GHS/QoL differed among CCI groups, with those with higher CCI scores (3+) reporting lower baseline GHS/QoL. However, for each CCI subgroup, there was a numerical non‐clinically meaningful improvement in GHS/QoL over the course of 18 months. Importantly, there were no clinically meaningful differences in GHS/QoL between the CCI 0 and CCI 1–2 groups, which comprised most of the POLARIS patient population (84.7%). While the effect of subpopulation idiosyncrasies and censoring patients who progressed or died, as well as of other factors, needs to be considered when interpreting results, extensive supplemental analyses revealed no tangible or obvious evidence of subpopulation selection or attrition bias [36]. Our results complement QoL findings of PALOMA‐2 and PALOMA‐3, wherein QoL was maintained for patients receiving palbociclib plus ET [37, 38]. Although QoL measurement is now a standard component of RCTs, real‐world data on QoL using PROs in ABC are more limited. The prospective design of the POLARIS study provides a comprehensive longitudinal assessment of HRQoL in the real‐world setting, which was maintained over 18 months for the overall cohort of patients while receiving palbociclib plus ET. Taken together with the findings herein, the available evidence indicates that HRQoL is preserved when palbociclib is added to ET in patients with HR+/HER2− ABC/MBC, including for those with a high comorbidity burden.

Assessment of palbociclib treatment patterns revealed a trend toward reduced initial doses, more dose modifications, and more dose reductions for patients with higher CCI scores in the 1 L setting. The initial dose data are consistent with the P‐REALITY X real‐world study, which found that patients with a higher mean National Cancer Institute comorbidity index score were more likely to start palbociclib at a lower dose [39]. In the ≥ 2 L setting, patients with higher CCI scores were also more likely to have reduced initial doses and more dosing interruptions. Although 125 mg/day is the label‐recommended initial dose for palbociclib [27], evidence indicates that efficacy is maintained when dose modifications aimed at managing toxicity are implemented [40]. Patients with blood and lymphatic disorders were also more likely to initiate palbociclib at a lower dose and had worse clinical outcomes than patients with other comorbid disorders. It has been reported that patients who started at a lower dose of palbociclib had worse survival outcomes than those who started at the recommended dose [41]. Of note, neutropenia and leukopenia are commonly reported adverse events (AEs) with palbociclib, [11, 12] and palbociclib dose reduction is a well‐established strategy for managing these AEs [27]. Although dosing adjustments have not been found to adversely affect survival outcomes in patients treated with palbociclib [40, 42, 43], these results in the context of blood and lymphatic comorbidities warrant future research.

To our knowledge, this is the first study to report a comprehensive set of real‐world clinical and HRQoL outcomes for patients with HR+/HER2− ABC/MBC and comorbidities. It had a large sample size and included patient records from > 100 academic and community institutions across North America. The relatively long follow‐up duration of approximately 3 years permitted the collection of mature OS data for most subgroups. The primary POLARIS study [23] recently reported clinical outcomes for the overall population that were broadly consistent with prior RCTs [11, 12, 44, 45] and other real‐world studies [21, 46, 47], thus supporting the generalizability of this POLARIS comorbidity subgroup analysis.

Nevertheless, interpretation of the data herein is subject to some limitations. Patient selection and treatment were determined by treating clinicians, and causality between treatments and outcomes could not be established. Outcomes across comorbidity subgroups may be confounded by other baseline patient characteristics. Some subgroups had small sample sizes and missing data over time. In contrast to RCTs, disease progression was not assessed based on Response Evaluation Criteria in Solid Tumors and was contingent on the treating clinician's interpretation of radiographic scans or pathology results. Classification of comorbidities was based on a history review, which may vary between institutions, investigators, and patients. Potential underreporting of comorbidities within charts may have led to an underestimation of the comorbidity burden. There was no ET‐only control arm in POLARIS, although this is common in non‐interventional studies. Finally, results from this analysis may not be generalizable to patients outside the United States and Canada.

## 5 Conclusions

Using real‐world data to assess clinical outcomes for populations with comorbid conditions that have been historically underrepresented in RCTs, we show that patients with HR+/HER2– ABC/MBC receiving palbociclib as 1 LOT or ≥ 2 LOT with CCI ≤ 2 score tended to have numerically longer rwPFS and OS than those with CCI 3+ score, and that on‐treatment GHS/QoL was maintained regardless of CCI score. Patients with CCI 0 had clinically meaningful and statistically significantly higher mean GHS/QoL scores than patients with CC3+ at each assessed time point. Additionally, patients with different common comorbidities had generally comparable clinical outcomes, except for those with blood and lymphatic system disorders, who tended to have shorter rwPFS and OS. Larger‐scale real‐world studies may be useful to further understand the impact of comorbidities on clinical outcomes.

## Author Contributions

**Debu Tripathy:** conceptualization (equal), formal analysis (equal), methodology (equal), resources (equal), writing – review and editing (equal). **Joanne L. Blum:** conceptualization (equal), formal analysis (equal), methodology (equal), resources (equal), writing – review and editing (equal). **Meghan S. Karuturi:** conceptualization (equal), formal analysis (equal), investigation (equal), methodology (equal), resources (equal), writing – review and editing (equal). **Steven McCune:** conceptualization (equal), data curation (equal), formal analysis (equal), investigation (equal), methodology (equal), resources (equal), writing – review and editing (equal). **Sobha Kurian:** formal analysis (equal), resources (equal), writing – review and editing (equal). **Mehdi M. Moezi:** data curation (equal), formal analysis (equal), investigation (equal), resources (equal), writing – review and editing (equal). **Daniel M. Anderson:** resources (equal), writing – review and editing (equal). **Yan Ji:** writing – review and editing (equal). **Timothy J. Pluard:** resources (equal), writing – review and editing (equal). **John Migas:** writing – review and editing (equal). **Shailendra Lakhanpal:** writing – review and editing (equal). **Erin Jepsen:** writing – review and editing (equal). **Yao Wang:** conceptualization (equal), data curation (equal), formal analysis (equal), investigation (equal), methodology (equal), resources (equal), writing – review and editing (equal). **Monica Z. Montelongo:** data curation (equal), formal analysis (equal), investigation (equal), writing – review and editing (equal). **Zhe Zhang:** formal analysis (equal), writing – review and editing (equal). **Joseph C. Cappelleri:** conceptualization (equal), data curation (equal), formal analysis (equal), investigation (equal), methodology (equal), writing – review and editing (equal). **Eric Gauthier:** conceptualization (equal), formal analysis (equal), methodology (equal), writing – review and editing (equal). **Gabrielle B. Rocque:** conceptualization (equal), formal analysis (equal), methodology (equal), resources (equal), writing – review and editing (equal).

## Funding

This work was supported by Pfizer Inc.

## Ethics Statement

The study was conducted in accordance with all applicable legal and regulatory requirements, the protection of patient personal data was ensured by all parties, and the study was prospectively approved by the appropriate institutional review boards or independent ethics committees.

## Conflicts of Interest

Debu Tripathy reports contracted research (clinical trials) paid to the institution from Novartis, Pfizer Inc, and Polyphor; and paid consultancy for AstraZeneca, OncoPep, GlaxoSmithKline, Gilead, Personalis, Sermonix, Puma, Pfizer Inc, Puma Biotechnology, Roche, Novartis, Menarini, BeiGene, AMBRX, and Jazz Pharmaceuticals. Joanne L. Blum has received consulting fees from Pfizer Inc and Tempus, and has participated in speakers bureaus for Pfizer Inc and Tempus. Steven McCune reports bureau fees from BMS and local PI nonfinancial interests (all institutional) from BMS, Merck, and Roche/Genentech. Yan Ji has received compensation for advisory boards: AstraZeneca, BMS. Timothy J. Pluard reports paid consultancies for Pfizer Inc, AstraZeneca, Jazz Pharmaceuticals, Daiichi Sankyo, and Gilead; research funding (institution) from Daiichi Sankyo, Jazz Pharmaceuticals, Pfizer Inc, Sermonix, Scorpion, Merck, DualityBio, Arvinas; speakers bureaus: Gilead, AstraZeneca, Stemline. John Migas reports contracted research (clinical trials) paid to the institution (Pfizer Inc, AstraZeneca, Jazz Pharmaceuticals). Erin Jepsen is employed by Novant Health Cancer Institute. Yao Wang, Zhe Zhang, Joseph C. Cappelleri, and Eric Gauthier are employees of and stockholders in Pfizer Inc. Monica Z. Montelongo is an employee of ICON, which was a paid consultant to Pfizer Inc in connection with the development of this manuscript. Gabrielle B. Rocque reports research funding from Genentech, Pfizer Inc, and Daiichi Sankyo; and consulting fees from Pfizer Inc and Gilead. She reports employment with Atlas Oncology Partners. Meghan S. Karuturi, Sobha Kurian, Mehdi M. Moezi, Daniel M. Anderson, and Shailendra Lakhanpal report no conflicts of interest.

## Supporting information


**Table S1:** Charlson Comorbidity Index scoring system.
**TABLE S2:** Baseline demographic and disease characteristics by comorbid disorder.
**TABLE S3:** EORTC QLQ‐C30 completion rate for GHS/QoL by CCI.
**TABLE S4:** Treatment patterns by comorbid disorder and LOT.
**TABLE S5:** Baseline demographic and disease characteristics by CCI, per‐label analysis set.
**TABLE S6:** Baseline demographic and disease characteristics by comorbid disorder, per‐label analysis set.
**FIGURE S1:** Palbociclib treatment patterns by CCI and LOT. CCI, Charlson Comorbidity Index; LOT, line of therapy.
**FIGURE S2:** rwPFS by CCI score in the 1 LOT (A) or ≥ 2 LOT (B) (per‐label). CCI, Charlson Comorbidity Index; CI, confidence interval; LOT, line of therapy; rwPFS, real‐world progression‐free survival.
**FIGURE S3:** OS by CCI score in the 1 LOT (A) or ≥ 2 LOT (B) (per‐label). CCI, Charlson Comorbidity Index; CI, confidence interval; LOT, line of therapy; NE, not estimable; NR, not reached; OS, overall survival.
**FIGURE S4:** rwPFS by comorbid disorder in the 1 LOT (A) or ≥ 2 LOT (B) (per‐label). CI, confidence interval; LOT, line of therapy; rwPFS, real‐world progression‐free survival.
**FIGURE S5:** OS by comorbid disorder in the 1 LOT (A) or ≥ 2 LOT (B) (per‐label). CI, confidence interval; LOT, line of therapy; NE, not estimable; OS, overall survival.
**FIGURE S6:** Mean GHS/QoL scores by CCI (per‐label). CCI, Charlson Comorbidity Index; GHS, global health status; QoL, quality of life.
**FIGURE S7:** GHS/QoL scores, mean change from baseline over time by CCI (per‐label). Mean change from baseline based on all available subjects who had data at both baseline and at Months 6, 12, and 18. Month 6, sample sizes: CCI 0, 146; CCI 1–2, 301; CCI ≥ 3, 83. Month 12, sample sizes: CCI 0, 107; CCI 1–2, 197; CCI ≥ 3, 50. Month 18, sample sizes: CCI 0, 73; CCI 1–2, 145; CCI ≥ 3, 40. CCI, Charlson Comorbidity Index; GHS, global health status; QoL, quality of life.

## Data Availability

Upon request, and subject to review, Pfizer will provide the data that support the findings of this study. Subject to certain criteria, conditions, and exceptions, Pfizer may also provide access to the related individual de‐identified participant data. See https://www.pfizer.com/science/clinical‐trials/data‐and‐results for more information.
