# Supplementary material for: Impact of Comorbidities on Clinical Outcomes and Quality of Life of Patients With Hormone Receptor‐Positive/Human Epidermal Growth Factor Receptor 2‐Negative (HR+/HER2−) Advanced Breast Cancer Treated With Palbociclib in the POLARIS Study
Source: Cancer Med. 2026 Apr 22;15(4):e71788. doi: 10.1002/cam4.71788 (PMC13101646; doi:10.1002/cam4.71788)

# Supplemental Materials

**Table S1** Charlson Comorbidity Index scoring system.

| **Comorbidity** | **Point score^a^** | **Common comorbid disorder category^b^** |
| --- | --- | --- |
| Cerebrovascular disease | 1 |  |
| Chronic pulmonary disease | 1 |  |
| Congestive heart failure | 1 | Cardiovascular |
| Connective tissue disease | 1 |  |
| Dementia | 1 |  |
| Diabetes with end organ damage | 2 | Metabolic and nutritional |
| Diabetes without end organ damage | 1 | Metabolic and nutritional |
| Depression | 1 | Psychiatric |
| Hemiplegia or paraplegia | 2 |  |
| History of myocardial infarction | 1 | Cardiovascular |
| HIV/AIDS | 6 |  |
| Hypertension | 1 | Cardiovascular |
| Mild liver disease | 1 |  |
| Moderate-to-severe liver disease | 3 |  |
| Use of warfarin | 1 |  |
| Moderate-to-severe renal disease | 2 |  |
| Peptic ulcer disease | 1 |  |
| Peripheral vascular disease | 1 | Cardiovascular |
| Skin ulcers/cellulitis | 2 |  |

Abbreviations: AIDS, acquired immune deficiency syndrome; CCI, Charlson Comorbidity Index; HIV, human immunodeficiency virus.

^a^Point scores were summed to generate an overall score of disease burden for each individual patient.

^b^Patients may have had other comorbidities beyond those listed here that were not applicable to the Charlson Comorbidity Index calculations; for example, blood and lymphatic system disorders such as anemia and neutropenia were common among the study population but did not contribute to comorbidity burden as assessed by CCI.

**TABLE S2** Baseline demographic and disease characteristics by comorbid disorder.

|  | **Cardiovascular disorders (*n = 690*)** | **Psychiatric disorders (*n = 332*)** | **Blood and lymphatic system disorders (*n = 230*)** | **Metabolic and nutritional disorders (*n = 228*)** |
| --- | --- | --- | --- | --- |
| Age at study enrollment, years |  |  |  |  |
| Mean (SD) | 67.1 (10.8) | 61.6 (12.7) | 63.4 (13.0) | 66.4 (10.9) |
| Median (range) | 68.0 (33–97) | 62.0 (27–90) | 65.5 (28–92) | 66.0 (40–88) |
| Female sex, No. (%) | 681 (98.7) | 328 (98.8) | 227 (98.7) | 223 (97.8) |
| Race, No. (%) |  |  |  |  |
| White | 570 (82.6) | 287 (86.4) | 171 (74.3) | 176 (77.2) |
| Black or African American | 89 (12.9) | 29 (8.7) | 48 (20.9) | 42 (18.4) |
| Asian | 8 (1.2) | 3 (0.9) | 5 (2.2) | 3 (1.3) |
| American Indian or Alaska Native | 5 (0.7) | 2 (0.6) | 1 (0.4) | 1 (0.4) |
| Native Hawaiian or other Pacific Islander | 1 (0.1) | 2 (0.6) | 1 (0.4) | 1 (0.4) |
| Other | 5 (0.7) | 5 (1.5) | 1 (0.4) | 1 (0.4) |
| Not reported or missing | 12 (1.7) | 4 (1.2) | 3 (1.3) | 4 (1.8) |
| Ethnicity, No. (%) |  |  |  |  |
| Not Hispanic or Latino | 617 (89.4) | 297 (89.5) | 202 (87.8) | 197 (86.4) |
| Hispanic or Latino | 55 (8.0) | 29 (8.7) | 25 (10.9) | 24 (10.5) |
| Not reported or missing | 18 (2.6) | 6 (1.8) | 3 (1.3) | 7 (3.1) |
| Time from ABC/MBC diagnosis, months |  |  |  |  |
| Mean (SD) | 14.1 (31.1) | 13.0 (27.6) | 15.5 (30.3) | 13.6 (33.1) |
| Median (range) | 1.3 (0–248) | 1.5 (0–248) | 2.0 (0–193) | 1.2 (0–242) |
| Disease stage at enrollment, No. (%) |  |  |  |  |
| Locally advanced (stage III) | 35 (5.1) | 19 (5.7) | 13 (5.7) | 16 (7.0) |
| Metastatic (stage IV) | 653 (94.6) | 313 (94.3) | 217 (94.3) | 211 (92.5) |
| Not reported | 2 (0.3) | 0 | 0 | 1 (0.4) |
| Disposition at enrollment, No. (%) |  |  |  |  |
| Recurrent from earlier stage (Stage 0-III) | 454 (65.8) | 247 (74.4) | 149 (64.8) | 158 (69.3) |
| De novo | 208 (30.1) | 71 (21.4) | 70 (30.4) | 59 (25.9) |
| Not reported | 28 (4.1) | 14 (4.2) | 11 (4.8) | 11 (4.8) |
| Bone metastases at MBC diagnosis,^a^ No. (%) |  |  |  |  |
| Bone plus other | 268 (41.0) | 110 (35.1) | 88 (40.6) | 88 (41.7) |
| Bone only | 222 (34.0) | 117 (37.4) | 70 (32.3) | 68 (32.2) |
| Visceral metastases^b^ at MBC diagnosis,^a^ No. (%) |  |  |  |  |
| Yes | 261 (40.0) | 125 (39.9) | 87 (40.1) | 95 (45.0) |
| No | 392 (60.0) | 188 (60.1) | 130 (59.9) | 116 (55.0) |

Abbreviations: ABC, advanced breast cancer; MBC, metastatic breast cancer; SD, standard deviation.

^a^Among patients with MBC at study enrollment.

^b^Visceral disease refers to metastases of the brain, liver, and/or lung/pleura.

**TABLE S3** EORTC QLQ-C30 completion rate for GHS/QoL by CCI.

|  |  | **CCI group** |  | **Total** |
| --- | --- | --- | --- | --- |
|  | **0** | **1–2** | **3+** |  |
| **Visit, No. complete (%^a^)** | ***n = 377*** | ***n = 682*** | ***n = 191*** | ***N = 1250*** |
| Baseline | 336 (89.1) | 651 (95.5) | 180 (94.2) | 1167 (93.4) |
| Month 6 | 219 (58.1) | 400 (58.7) | 113 (59.2) | 732 (58.6) |
| Month 12 | 151 (40.1) | 262 (38.4) | 71 (37.2) | 484 (38.7) |
| Month 18 | 103 (27.3) | 198 (29.0) | 52 (27.2) | 353 (28.2) |

*Note:* Month 6, eligible patients: CCI 0, 335; CCI 1–2, 595; CCI ≥3, 170; Total, 1100
Month 12, eligible patients: CCI 0, 311; CCI 1–2, 527; CCI ≥3, 140; Total, 978
Month 18, eligible patients: CCI 0, 262; CCI 1–2, 453; CCI ≥3, 119; Total, 834

Abbreviations: CCI, Charlson Comorbidity Index; EORTC QLQ-30, European Organisation for Research and Treatment of Cancer Quality of Life Questionnaire Core 30; GHS, global health status; QoL, quality of life.

^a^Percentage calculated by dividing number of patients who completed the global health status/QoL items by the total number of patients in each category who received at least 1 dose of palbociclib.

**TABLE S4** Treatment patterns by comorbid disorder and LOT.

| **1LOT (*n = 901*)** |  |  |  |  |  |
| --- | --- | --- | --- | --- | --- |
| **Comorbid disorder^a^** |  | **Cardiovascular disorders** | **Psychiatric disorders** | **Blood and lymphatic system disorders** | **Metabolic and nutritional disorders** |
|  | **No.** | ***n = 501*** | ***n = 236*** | ***n = 144*** | ***n = 172*** |
| Starting dose, No. (%) |  |  |  |  |  |
| 125 mg | 827 | 459 (91.6) | 213 (90.3) | 121 (84.0) | 153 (89.0) |
| 100 mg | 53 | 33 (6.6) | 18 (7.6) | 17 (11.8) | 17 (9.9) |
| 75 mg | 21 | 9 (1.8) | 5 (2.1) | 6 (4.2) | 2 (1.2) |
| Dose modification, No. (%) |  |  |  |  |  |
| No | 551 | 296 (59.1) | 147 (62.3) | 85 (59.0) | 107 (62.2) |
| ≥1 | 350 | 205 (40.9) | 89 (37.7) | 59 (41.0) | 65 (37.8) |
| Dose decrease, No. (%) |  |  |  |  |  |
| No | 559 | 300 (59.9) | 150 (63.6) | 87 (60.4) | 109 (63.4) |
| ≥1 | 342 | 201 (40.1) | 86 (36.4) | 57 (39.6) | 63 (36.6) |
| Dosing interruption, No. (%) |  |  |  |  |  |
| No | 761 | 428 (85.4) | 203 (86.0) | 126 (87.5) | 144 (83.7) |
| ≥1 | 140 | 73 (14.6) | 33 (14.0) | 18 (12.5) | 28 (16.3) |
|  |  |  |  |  |  |
| **≥2LOT (*n = 349*)** |  |  |  |  |  |
| **Comorbid disorder^a^** |  | **Cardiovascular disorders** | **Psychiatric disorders** | **Blood and lymphatic system disorders** | **Metabolic and nutritional disorders** |
|  |  | ***n = 189*** | ***n = 96*** | ***n = 86*** | ***n = 56*** |
| Starting dose, No. (%) |  |  |  |  |  |
| 125 mg | 297 | 161 (85.2) | 86 (89.6) | 67 (77.9) | 46 (82.1) |
| 100 mg | 39 | 21 (11.1) | 4 (4.2) | 15 (17.4) | 9 (16.1) |
| 75 mg | 13 | 7 (3.7) | 6 (6.3) | 4 (4.7) | 1 (1.8) |
| Dose modification, No. (%) |  |  |  |  |  |
| No | 223 | 123 (65.1) | 64 (66.7) | 59 (68.6) | 39 (69.6) |
| ≥1 | 126 | 66 (34.9) | 32 (33.3) | 27 (31.4) | 17 (30.4) |
| Dose decrease, No. (%) |  |  |  |  |  |
| No | 227 | 123 (65.1) | 65 (67.7) | 60 (69.8) | 39 (69.6) |
| ≥1 | 122 | 66 (34.9) | 31 (32.3) | 26 (30.2) | 17 (30.4) |
| Dosing interruption, No. (%) |  |  |  |  |  |
| No | 300 | 163 (86.2) | 82 (85.4) | 73 (84.9) | 47 (83.9) |
| ≥1 | 49 | 26 (13.8) | 14 (14.6) | 13 (15.1) | 9 (16.1) |

Abbreviation: LOT, line of therapy.

^a^Owing to some patients meeting the criteria for multiple comorbid disorders, individual disorder category sample sizes may summate to a number greater than the number of patients in each LOT.

**TABLE S5** Baseline demographic and disease characteristics by CCI, per label analysis set.

|  | **CCI 0 (*n = 243*)** | **CCI 1–2 (*n = 485*)** | **CCI 3+ (*n = 133*)** | **Total (*N = 861*)** |
| --- | --- | --- | --- | --- |
| Age at study enrollment, years |  |  |  |  |
| No. (missing) | 243 (0) | 484 (1) | 133 (0) | 860 (1) |
| Mean (SD) | 58.5 (12.3) | 65.3 (11.8) | 67.2 (10.2) | 63.7 (12.1) |
| Median (range) | 60.0 (22–97) | 66.0 (29–97) | 68.0 (43–86) | 64.0 (22–97) |
| Female sex, No. (%) | 241 (99.2) | 477 (98.4) | 131 (98.5) | 849 (98.6) |
| Race, No. (%) |  |  |  |  |
| White | 192 (79.0) | 408 (84.1) | 109 (82.0) | 709 (82.3) |
| Black or African American | 26 (10.7) | 54 (11.1) | 19 (14.3) | 99 (11.5) |
| Asian | 6 (2.5) | 4 (0.8) | 1 (0.8) | 11 (1.3) |
| American Indian or Alaska Native | 1 (0.4) | 3 (0.6) | 2 (1.5) | 6 (0.7) |
| Native Hawaiian or other Pacific Islander | 1 (0.4) | 2 (0.4) | 0 | 3 (0.3) |
| Other | 8 (3.3) | 6 (1.2) | 1 (0.8) | 15 (1.7) |
| Not reported/missing | 9 (3.7) | 8 (1.6) | 1 (0.8) | 18 (2.1) |
| Ethnicity, No. (%) |  |  |  |  |
| Not Hispanic or Latino | 218 (89.7) | 434 (89.5) | 123 (92.5) | 775 (90.0) |
| Hispanic or Latino | 16 (6.6) | 36 (7.4) | 9 (6.8) | 61 (7.1) |
| Not reported/missing | 9 (3.7) | 15 (3.1) | 1 (0.8) | 25 (2.9) |
| Time since ABC/MBC diagnosis, months |  |  |  |  |
| No. (missing) | 243 (0) | 481 (4) | 133 (0) | 857 (4) |
| Mean (SD) | 7.8 (22.2) | 10.0 (23.1) | 10.4 (22.6) | 9.4 (22.8) |
| Median (range) | 1.1 (0–191) | 1.0 (0–193) | 1.2 (0–131) | 1.1 (0–193) |
| Disease stage at enrollment, No. (%) |  |  |  |  |
| Locally advanced (stage III) | 10 (4.1) | 21 (4.3) | 6 (4.5) | 37 (4.3) |
| Metastatic (stage IV) | 233 (95.9) | 462 (95.3) | 127 (95.5) | 822 (95.5) |
| Not reported | 0 | 2 (0.4) | 0 | 2 (0.2) |
| Disposition at enrollment, No. (%) |  |  |  |  |
| Recurrent from earlier stage | 167 (68.7) | 336 (69.3) | 86 (64.7) | 589 (68.4) |
| De novo, newly diagnosed stage IV | 64 (26.3) | 131 (27.0) | 42 (31.6) | 237 (27.5) |
| Not reported | 12 (4.9) | 18 (3.7) | 5 (3.8) | 35 (4.1) |
| Bone metastases at MBC diagnosis,^a^ No. (%) |  |  |  |  |
| Bone plus other | 92 (39.5) | 181 (39.2) | 58 (45.7) | 331 (40.3) |
| Bone only | 77 (33.0) | 181 (39.2) | 41 (32.3) | 299 (36.4) |
| Visceral metastases^b^ at MBC diagnosis,^a^ No. (%) |  |  |  |  |
| Yes | 99 (42.5) | 179 (38.7) | 53 (41.7) | 331 (40.3) |
| No | 134 (57.5) | 283 (61.3) | 74 (58.3) | 491 (59.7) |

Abbreviations: ABC, advanced breast cancer; CCI, Charlson Comorbidity Index; MBC, metastatic breast cancer; SD, standard deviation.

^a^Among patients with MBC at study enrollment.

^b^Visceral disease refers to metastases of the brain, liver, and/or lung/pleura.

**TABLE S6** Baseline demographic and disease characteristics by comorbid disorder, per label analysis set.

|  | **Cardiovascular disorders (*n = 492*)** | **Psychiatric disorders (*n = 231*)** | **Blood and lymphatic  system disorders (*n = 145*)** | **Metabolic and nutritional disorders  (*n = 155*)** |
| --- | --- | --- | --- | --- |
| Age at study enrollment, years |  |  |  |  |
| No. (missing) | 491 (1) | 230 (1) | 145 (0) | 155 (0) |
| Mean (SD) | 66.9 (10.7) | 62.3 (12.1) | 64.3 (12.0) | 65.9 (10.7) |
| Median (range) | 68.0 (33–97) | 63.0 (29–90) | 65.0 (28–92) | 66.0 (43–88) |
| Female sex, No. (%) | 486 (98.8) | 227 (98.3) | 143 (98.6) | 152 (98.1) |
| Race, No. (%) |  |  |  |  |
| White | 407 (82.7) | 201 (87.0) | 110 (75.9) | 121 (78.1) |
| Black or African American | 66 (13.4) | 21 (9.1) | 32 (22.1) | 28 (18.1) |
| Asian | 3 (0.6) | 2 (0.9) | 1 (0.7) | 2 (1.3) |
| American Indian or Alaska Native | 5 (1.0) | 1 (0.4) | 0 | 1 (0.6) |
| Native Hawaiian or other Pacific Islander | 0 | 2 (0.9) | 0 | 0 |
| Other | 3 (0.6) | 3 (1.3) | 0 | 1 (0.6) |
| Not reported/missing | 8 (1.6) | 1 (0.4) | 2 (1.4) | 2 (1.3) |
| Ethnicity, No. (%) |  |  |  |  |
| Not Hispanic or Latino | 441 (89.6) | 209 (90.5) | 133 (91.7) | 136 (87.7) |
| Hispanic or Latino | 35 (7.1) | 18 (7.8) | 11 (7.6) | 14 (9.0) |
| Not reported/missing | 16 (3.3) | 4 (1.7) | 1 (0.7) | 5 (3.2) |
| Time since ABC/MBC diagnosis, months |  |  |  |  |
| No. (missing) | 489 (3) | 231 (0) | 145 (0) | 153 (2) |
| Mean (SD) | 10.2 (23.8) | 9.2 (19.1) | 12.9 (28.9) | 8.4 (21.2) |
| Median (range) | 1.1 (0–193) | 1.2 (0–94) | 1.4 (0–193) | 1.0 (0–131) |
| Disease stage at enrollment, No. (%) |  |  |  |  |
| Locally advanced (stage III) | 22 (4.5) | 14 (6.1) | 3 (2.1) | 9 (5.8) |
| Metastatic (stage IV) | 468 (95.1) | 217 (93.9) | 142 (97.9) | 145 (93.5) |
| Not reported | 2 (0.4) | 0 | 0 | 1 (0.6) |
| Disposition at enrollment, No. (%) |  |  |  |  |
| Recurrent from earlier stage | 323 (65.7) | 172 (74.5) | 93 (64.1) | 111 (71.6) |
| De novo, newly diagnosed stage IV | 152 (30.9) | 48 (20.8) | 49 (33.8) | 39 (25.2) |
| Not reported | 17 (3.5) | 11 (4.8) | 3 (2.1) | 5 (3.2) |
| Bone metastases at MBC diagnosis,^a^ No. (%) |  |  |  |  |
| Bone plus other | 194 (41.5) | 81 (37.3) | 62 (43.7) | 63 (43.4) |
| Bone only | 174 (37.2) | 84 (38.7) | 48 (33.8) | 54 (37.2) |
| Visceral metastases^b^ at MBC diagnosis,^a^ No. (%) |  |  |  |  |
| Yes | 177 (37.8) | 86 (39.6) | 57 (40.1) | 62 (42.8) |
| No | 291 (62.2) | 131 (60.4) | 85 (59.9) | 83 (57.2) |

Abbreviations: ABC, advanced breast cancer; MBC, metastatic breast cancer; SD, standard deviation.

^a^Among patients with MBC disease at study enrollment.

^b^Visceral disease refers to metastases of the brain, liver, and/or lung/pleura.

**FIGURE S1**. Palbociclib treatment patterns by CCI and LOT. CCI, Charlson Comorbidity Index; LOT, line of therapy.


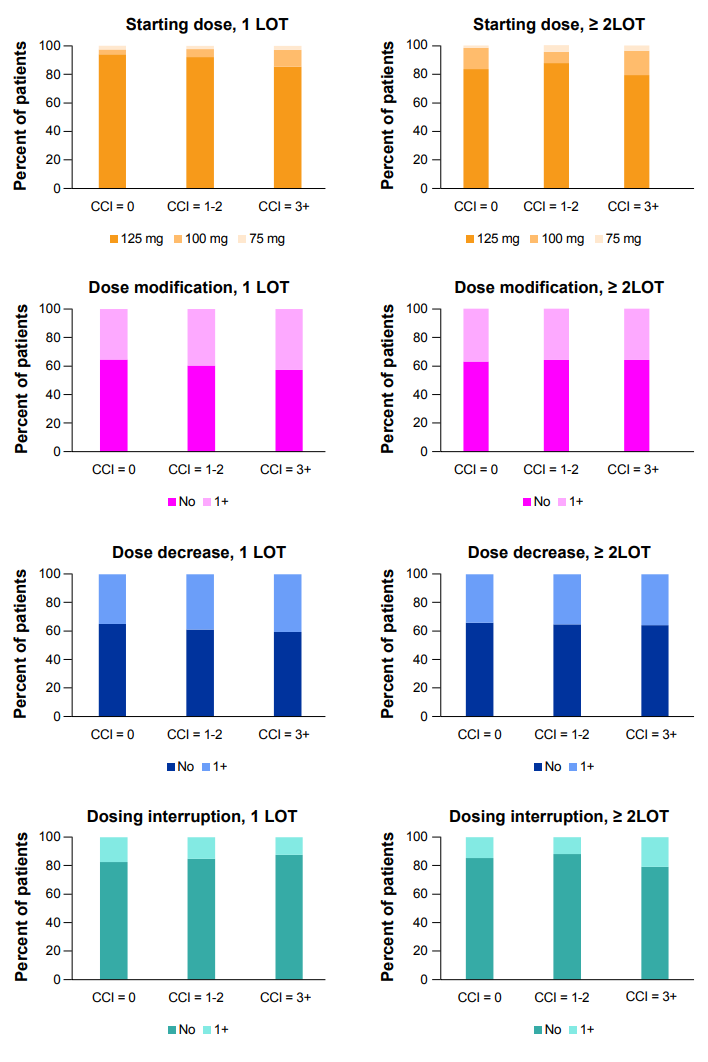


**FIGURE S2** rwPFS by CCI score in the 1LOT (A) or ≥2LOT (B) (per-label). CCI, Charlson Comorbidity Index; CI, confidence interval; LOT, line of therapy; rwPFS, real-world progression-free survival.


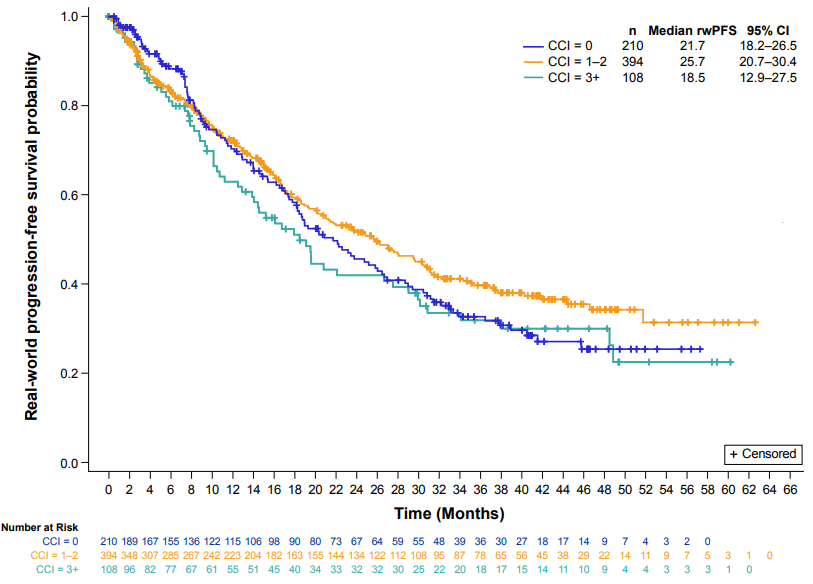
A


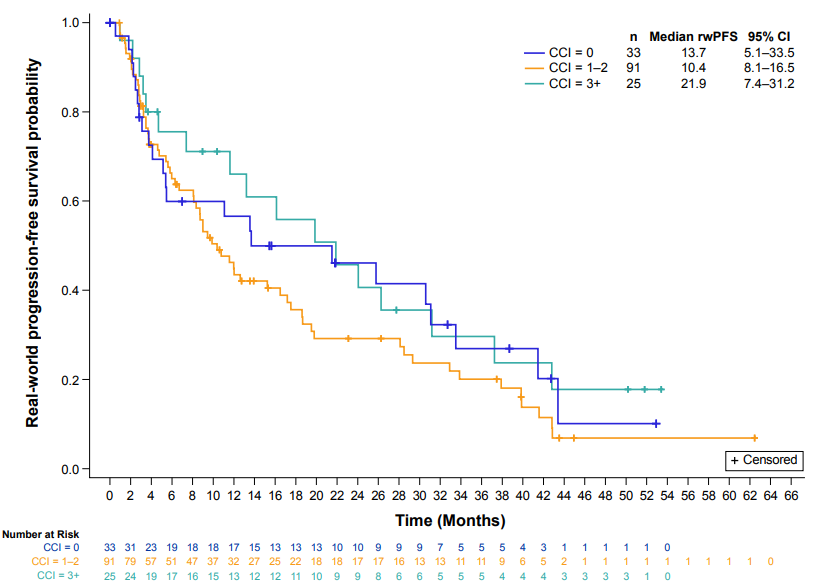


B

**FIGURE S3** OS by CCI score in the 1LOT (A) or ≥2LOT (B) (per-label). CCI, Charlson Comorbidity Index; CI, confidence interval LOT, line of therapy; NE, not estimable; NR, not reached; OS, overall survival.

A


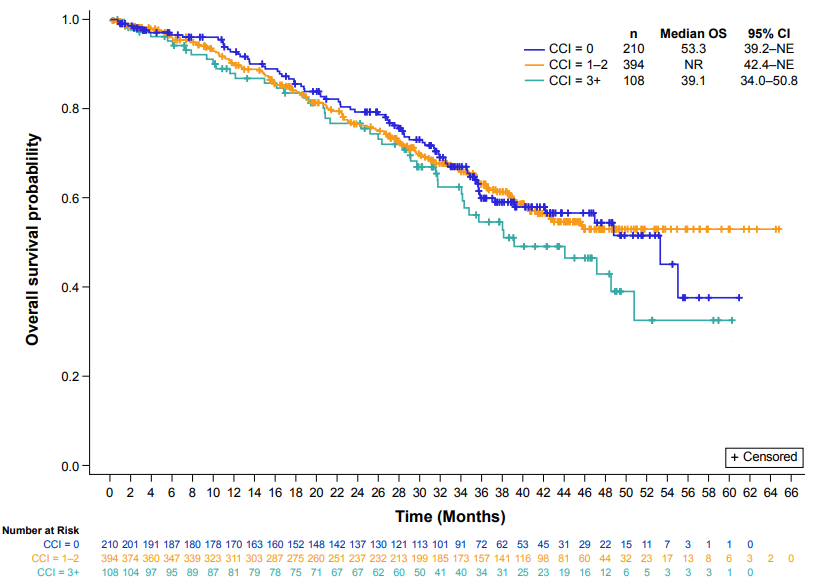


B


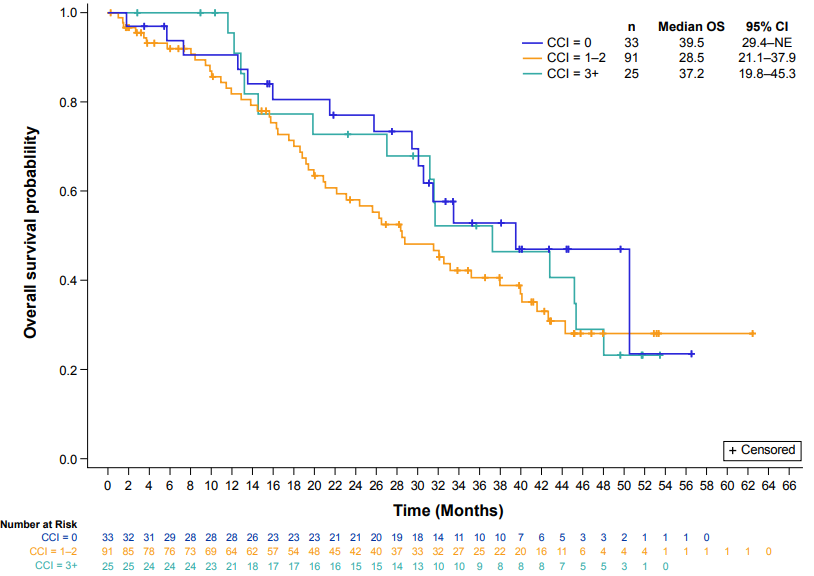


**FIGURE S4** rwPFS by comorbid disorder in the 1LOT (A) or ≥2LOT (B) (per-label). CI, confidence interval; LOT, line of therapy; rwPFS, real-world progression-free survival.

A


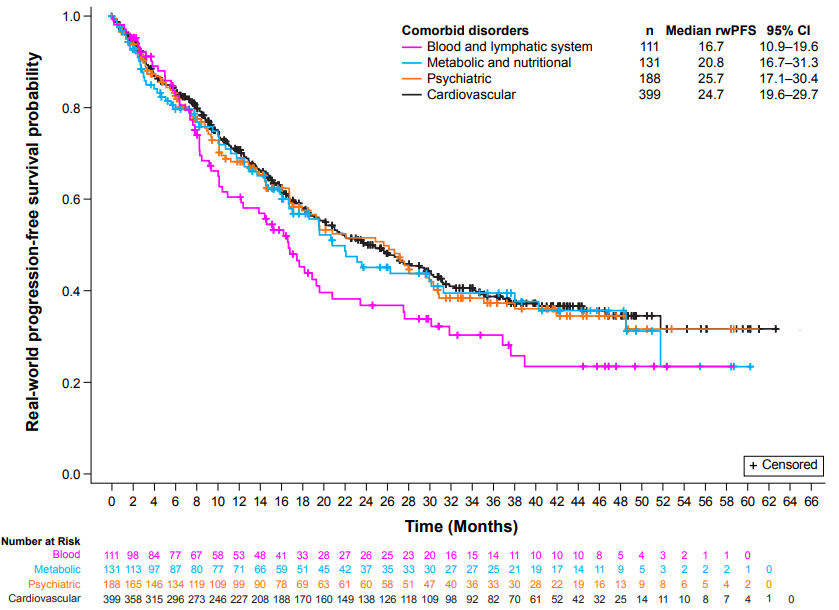


B


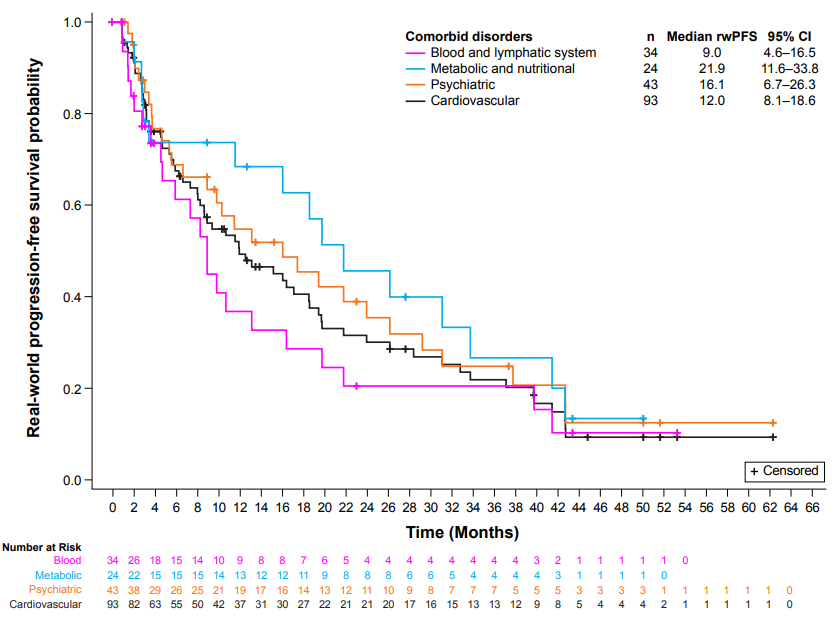


**FIGURES S5** OS by comorbid disorder in the 1LOT (A) or ≥2LOT (B) (per-label). CI, confidence interval; LOT, line of therapy; NE, not estimable; OS, overall survival.

A


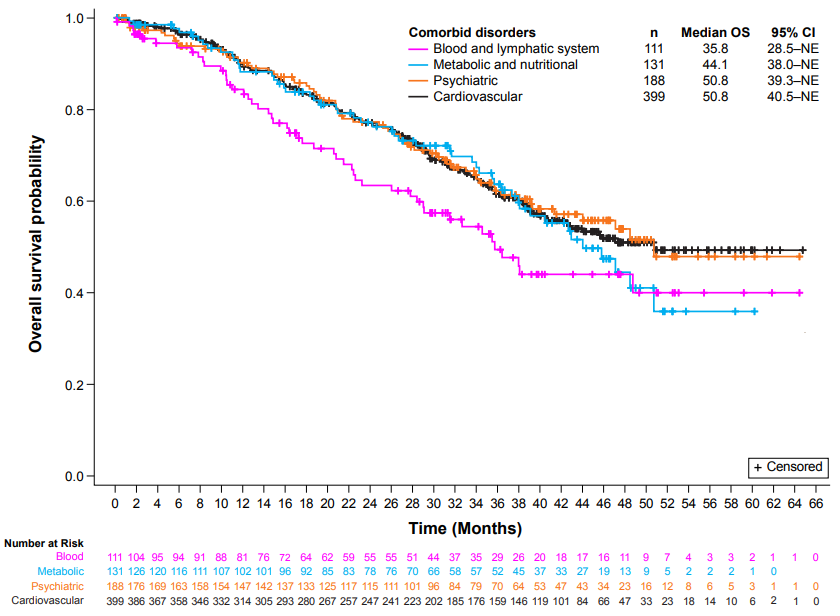


B


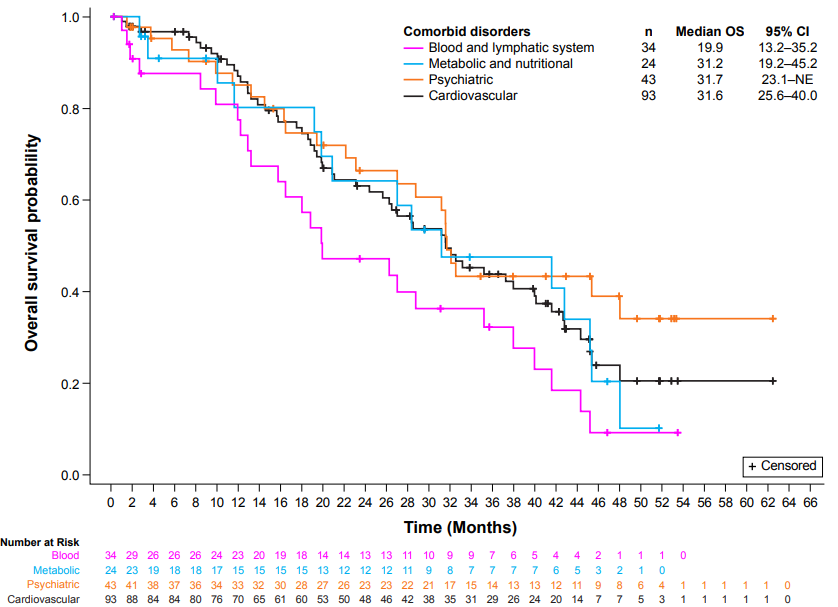


**FIGURE S6** Mean GHS/QoL scores by CCI (per-label). CCI, Charlson Comorbidity Index; GHS, global health status; QoL, quality of life.


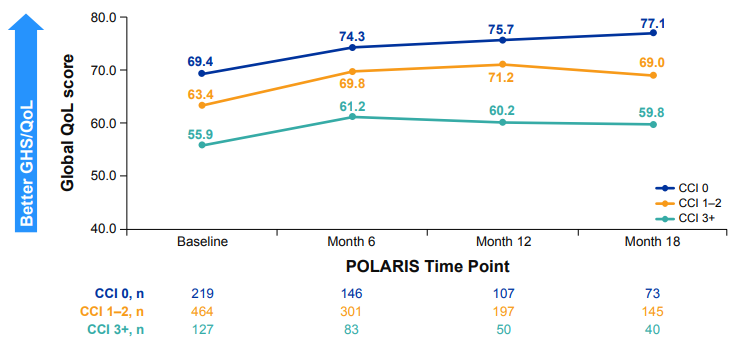


**FIGURE S7** GHS/QoL scores, mean change from baseline over time by CCI (per-label). Mean change from baseline based on all available subjects who had data at both baseline and at Months 6, 12, and 18. Month 6, sample sizes: CCI 0, 146; CCI 1–2, 301; CCI ≥3, 83.
Month 12, sample sizes: CCI 0, 107; CCI 1–2, 197; CCI ≥3, 50. Month 18, sample sizes: CCI 0, 73; CCI 1–2, 145; CC1 ≥3, 40. CCI, Charlson Comorbidity Index; GHS, global health status; QoL, quality of life.


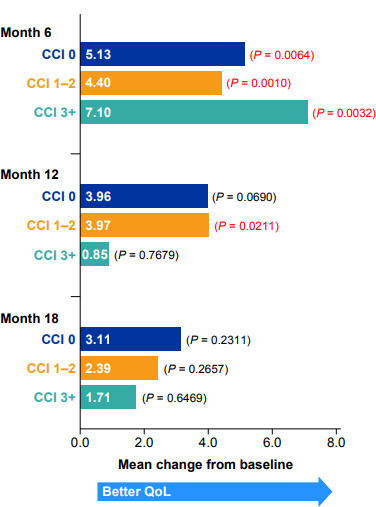

Supplement: Supplementary file 1 — Table S1: Charlson Comorbidity Index scoring system. TABLE S2: Baseline demographic and disease characteristics by comorbid disorder. TABLE S3: EORTC QLQ‐C30 completion rate for GHS/QoL by CCI. TABLE S4: Treatment patterns by comorbid disorder and LOT. TABLE S5: Baseline demographic and disease characteristics by CCI, per‐label analysis set. TABLE S6: Baseline demographic and disease characteristics by comorbid disorder, per‐label analysis set. FIGURE S1: Palbociclib treatment patterns by CCI and LOT. CCI, Charlson Comorbidity Index; LOT, line of therapy. FIGURE S2: rwPFS by CCI score in the 1 LOT (A) or ≥ 2 LOT (B) (per‐label). CCI, Charlson Comorbidity Index; CI, confidence interval; LOT, line of therapy; rwPFS, real‐world progression‐free survival. FIGURE S3: OS by CCI score in the 1 LOT (A) or ≥ 2 LOT (B) (per‐label). CCI, Charlson Comorbidity Index; CI, confidence interval; LOT, line of therapy; NE, not estimable; NR, not reached; OS, overall survival. FIGURE S4: rwPFS by comorbid disorder in the 1 LOT (A) or ≥ 2 LOT (B) (per‐label). CI, confidence interval; LOT, line of therapy; rwPFS, real‐world progression‐free survival. FIGURE S5: OS by comorbid disorder in the 1 LOT (A) or ≥ 2 LOT (B) (per‐label). CI, confidence interval; LOT, line of therapy; NE, not estimable; OS, overall survival. FIGURE S6: Mean GHS/QoL scores by CCI (per‐label). CCI, Charlson Comorbidity Index; GHS, global health status; QoL, quality of life. FIGURE S7: GHS/QoL scores, mean change from baseline over time by CCI (per‐label). Mean change from baseline based on all available subjects who had data at both baseline and at Months 6, 12, and 18. Month 6, sample sizes: CCI 0, 146; CCI 1–2, 301; CCI ≥ 3, 83. Month 12, sample sizes: CCI 0, 107; CCI 1–2, 197; CCI ≥ 3, 50. Month 18, sample sizes: CCI 0, 73; CCI 1–2, 145; CCI ≥ 3, 40. CCI, Charlson Comorbidity Index; GHS, global health status; QoL, quality of life. [file CAM4-15-e71788-s001.docx]
